# New Properties of Holomorphic Sobolev–Hardy Spaces

**DOI:** 10.1007/s11785-024-01637-8

**Published:** 2024-12-13

**Authors:** William Gryc, Loredana Lanzani, Jue Xiong, Yuan Zhang

**Affiliations:** 1 Department of Mathematics and Computer Science, Muhlenberg College, Allentown, PA 18104 USA; 2 Department of Mathematics, Syracuse University, Syracuse, NY 13244 USA; 3 Department of Mathematics, University of Bologna, Bologna, Italy; 4 Department of Mathematics, University of Colorado, Boulder, CO 80309 USA; 5 Department of Mathematical Sciences, Purdue University Fort Wayne, Fort Wayne, IN 46805-1499 USA

**Keywords:** Lipschitz domain, Sobolev–Hardy space, Neumann problem, $\bar{\partial }$, Reproducing kernel Hilbert space, 30H10, 30E20, 30E25, 31A25

## Abstract

We give new characterizations of the optimal data space for the $L^p(bD,\sigma )$-Neumann boundary value problem for the $\bar{\partial }$ operator associated to a bounded, Lipschitz domain $D\subset \mathbb {C}$. We show that the solution space is embedded (as a Banach space) in the Dirichlet space and that for $p=2$, the solution space is a reproducing kernel Hilbert space.

## Introduction

Let $D$ be a bounded Lipschitz domain in $\mathbb C$ whose boundary $bD$ is endowed with the induced Lebesgue measure $\sigma$. Let $\mathcal {H}^p(D)$ be the **holomorphic Hardy space**:

$$\begin{aligned} \mathcal {H}^p(D):= \{F\in \vartheta (D):\, F^*\in L^p(bD, \sigma )\},\ \quad 0<p\le \infty \end{aligned}$$

with $\vartheta (D)$ denoting the set of holomorphic functions on $D$ and $F^*$ the non-tangential maximal function of *F*. It is well-known that if $D$ is simply connected, every element *F* of $\mathcal {H}^p(D)$ admits a nontangential limit $\dot{F}$ that lies in $L^p(bD,\sigma )$ (see [5, Theorem 10.3]). On the other hand, since Lipschitz domains are local epigraphs, any bounded Lipschitz domain must be finitely connected. Hence, an elementary localization argument shows that any $F\in \mathcal {H}^p(D)$ has a nontangential limit $\dot{F}$ defined $\sigma$-a.e. on $bD$. We will call the set of all such nontangential limits $h^p(bD)$. That is,

$$\begin{aligned} h^p(bD) := \left\{ \dot{F}\ :\ F\in \mathcal {H}^p(D)\right\} \, \subsetneq \, L^p(bD, \sigma ). \end{aligned}$$

Let $\mathcal {H}^{1, p}(D)$ be the **holomorphic Sobolev–Hardy space**

$$\begin{aligned} \mathcal {H}^{1, p}(D): = \{G\in {\vartheta }(D): G'\in \mathcal {H}^p(D)\},\quad p>0. \end{aligned}$$

It is shown in [6] that, given $g\in L^p(bD, \sigma )$ subject to the compatibility condition: $\displaystyle {\int \limits _{bD}g\, d\sigma =0}$, the **Neumann problem for the **$\overline{\partial }$**operator**

1 $$\begin{aligned} \left\{ \begin{array}{ll} \bar{\partial }G = 0 &  \text {in} \ \ D;\\ \\ \displaystyle {\frac{\partial G}{\partial n}(\zeta )} = g(\zeta ) &  \text {for}\ \sigma \text {-a.e.}\ \zeta \in bD; \\ \\ (G')^*\in L^p(bD, \sigma )&  \end{array} \right. \end{aligned}$$

is solvable if and only if the data *g* belongs to

2 $$\begin{aligned} \mathfrak {n}^{p}(bD):= \left\{ -iT(\zeta )\dot{(G')}(\zeta ): \ G\in \mathcal H^{1, p}(D)\right\} ,\quad 1\le p\le \infty , \end{aligned}$$

where $\zeta \mapsto T(\zeta )$ is the unit tangent vector field for $bD$. Moreover, if $g\in \mathfrak {n}^{p}(bD)$ then all solutions of (1) belong to $\mathcal {H}^{1, p}(D)$. Any two solutions of (1) differ by an additive constant, hence for any fixed $\alpha \in D$ the space

$$\begin{aligned} \mathcal {H}_{\alpha }^{1, p}(D):= \{F\in \mathcal {H}^{1,p}(D): F(\alpha )=0\} \end{aligned}$$

contains precisely one solution of (1). In the case when $p=2$ and $D$ is simply-connected, $\mathcal {H}_{\alpha }^{1, 2}(D)$ is a Hilbert space with inner product

$$\begin{aligned} \langle F, G\rangle _{\mathcal {H}_{\alpha }^{1, 2}(D)} := \int \limits _{bD}\!\! \dot{(F')}(\zeta ) \,\overline{\dot{(G')}(\zeta )}\,d\sigma (\zeta ). \end{aligned}$$

In this paper we explore properties of $\mathcal {H}_\alpha ^{1, 2}(bD)$ and of $\mathfrak {n}^{p}(bD)$. Specifically, after recalling a few well-known basic properties of Lipschitz domains (Sect. 2), we show that the solution space $\mathcal {H}_{\alpha }^{1,2}(D)$ is a reproducing kernel Hilbert space (Theorem 3.1) and for $D=\mathbb {D}$ (the unit disc) we compute its reproducing kernel. Next we show that for $1<p<\infty$ there is a Banach space embedding of $\mathcal {H}_{\alpha }^{1,p}(D)$ in the Dirichlet space $\mathcal {D}^p_\alpha (D)$ (Theorem 3.3). In Sect. 4 we give various characterizations of $\mathfrak {n}^p(bD)$ for simply connected $D$: in terms of $L^p(bD,\sigma )$-functions whose moments all vanish on $bD$; or in terms of the vanishing of the Cauchy integral over $\overline{D}^c$, the complement of the closure of $D$; as well as in terms of its conformal map (Theorems and 4.3). Finally, in Sect. 5 we provide a characterization of $\mathfrak {n}^p(bD)$ for multiply connected $D$: in this case the aforementioned vanishing moment condition takes a more restrictive form, see Theorem 5.3.

## Preliminaries

### Lipschitz Domains

Throughout this paper the domains under consideration will be Lipschitz domains on $\mathbb C$, as defined below.

#### Definition 2.1

A bounded domain $D\subset \mathbb {C}$ with boundary $bD$ is called a **Lipschitz domain** if there are finitely many rectangles $\{R_j\}_{j=1}^m$ with sides parallel to the coordinate axes, angles $\{\theta _j\}_{j=1}^m$, and Lipschitz functions $\phi _j:\mathbb {R}\rightarrow \mathbb {R}$ such that the collection $\{e^{-i\theta _j}R_j\}_{j=1}^m$ covers $bD$ and $(e^{i\theta _j}D)\cap R_j=\{x+iy: y > \phi _j(x),\ x\in (a_j, b_j)\}$ for some $a_j<b_j<\infty$. We refer to such $R_j$’s as *coordinate rectangles*.

#### Definition 2.2

Let $D$ be a bounded Lipschitz domain. For any $\zeta \in bD$, let $\{\Gamma (\zeta ), \zeta \in D\}$ be a family of truncated (one-sided) open cones $\Gamma (\zeta )$ with vertex at $\zeta$ satisfying the following property: for each rectangle $R_j$ in Definition 2.1, there exists two cones $\Delta _1$ and $\Delta _2$, each with vertex at the origin and axis along the *y* axis such that for $\zeta \in bD\cap e^{-i\theta _j}R_j$,

$$ e^{-i\theta _j}\Delta _1 +\zeta \quad \subset \quad \Gamma (\zeta )\quad \subset \quad \overline{\Gamma (\zeta )}\setminus \{\zeta \}\quad \subset \quad e^{-i\theta _j}\Delta _2+\zeta \quad \subset \quad D\ \cap \ e^{-i\theta _j}R_j. $$

It is well known that for Lipschitz $D$, $\Gamma (\zeta )\ne \emptyset$ for any $\zeta \in bD$; see e.g., [4] or [12, Section 0.4]. We will sometimes refer to $\Gamma (\zeta )$ as a *regular cone*, or a *coordinate cone*. For a function *F* on $D$ and $\zeta \in bD$, we define the **nontangential maximal function** $F^*(\zeta )$ and the **nontangential limit** $\dot{F}(\zeta )$ as

$$ F^*(\zeta ):=\sup \limits _{z\in \Gamma (\zeta )}|F(z)|\,, \qquad \text {and}\quad \dot{F}(\zeta )= \lim _{{\mathop { z\in \Gamma (\zeta )}\limits ^{z\rightarrow \zeta }}} F(z)\quad \text {if such limit exists.} $$

We will need an approximation scheme of $D$ by smooth subdomains constructed by Nečas in [10], which we refer to as a **Nečas exhaustion of **$D$. See also [8] and [12, Theorem 1.12]. (Recall that Lipschitz functions are differentiable almost everywhere; thus if $D$ is Lipschitz and simply connected its boundary $bD$ is a rectifiable Jordan curve that admits a (positively oriented) unit tangent vector $T(\zeta )$ as $\sigma$-a.e. $\zeta \in bD$.)

#### Lemma 2.3

[10, p. 5][12, Theorem 1.12] Let $D$ be a bounded Lipschitz domain. There exists a family $\{D_k\}_{k=1}^\infty$ of smooth domains with $D_k$ compactly contained in $D$ that satisfy the following:

- For each *k* there exists a Lipschitz diffeomorphism $\Lambda _k$ that takes $D$ to $D_k$ and extends to the boundaries: $\Lambda _k:bD\rightarrow bD_k$ with the property that
  $$\begin{aligned} \sup \{|\Lambda _k(\zeta )-\zeta |:\zeta \in bD\}\le C/k \end{aligned}$$
  for some fixed constant *C*. Moreover $\Lambda _k(\zeta )\in \Gamma (\zeta )$.
- There is a covering of $bD$ by finitely many coordinate rectangles which also form a family of coordinate rectangles for $bD_k$ for each *k*. Furthermore for every such rectangle *R*, if $\phi$ and $\phi _k$ denote the Lipschitz functions whose graphs describe the boundaries of $D$ and $D_k$, respectively, in *R*, then $\Vert (\phi _k)'\Vert _\infty \le \Vert \phi '\Vert _\infty$ for any *k*; $\phi _k\rightarrow \phi$ uniformly as $k\rightarrow \infty$, and $(\phi _k)'\rightarrow \phi '$ a.e. and in every $L^p((a, b))$ with $(a, b)\subset \mathbb R$ as in Definition 2.1.
- There exist constants $0<m<M<\infty$ and positive functions (Jacobians) $w_k:bD\rightarrow [m,M]$ for any $k\in \mathbb N$, such that for any measurable set $F\subseteq bD$ and for any measurable function $f_k$ on $\Lambda _k (F)$ the following change-of-variables formula holds:
  $$\int \limits _{F} \!\! f_k(\Lambda _k(\eta ))\,w_k(\zeta )\,d\sigma (\eta ) = \int \limits _{ \Lambda _k(F)} \!\!\! f_k(\eta _k)\,d\sigma _k(\eta _k).$$
  where $d\sigma _k$ denotes arc-length measure on $bD_k$. Furthermore we have
  $$w_k\rightarrow 1 \quad \sigma \text {-a.e.}\ bD\ \ \text {and in every}\quad L^p(bD,\sigma )\ \ \text {for any}\ \ 1\le p <\infty \,.$$
- Let $T_k$ denote the unit tangent vector for $bD_k$ and *T* denote the unit tangent vector of $bD$. We have that
  $$T_k\rightarrow T \quad \sigma \text {-a.e.}\ bD\ \ \text {and in every}\quad L^p(bD,\sigma )\ \ \text {for any}\ \ 1\le p <\infty \,.$$

Note that in conclusions *(b)* through *(d)* the exponent $p=\infty$ cannot be allowed unless $D$ is of class $C^1$. Nečas exhaustions can be used to transfer well-known results for holomorphic functions over domains with smooth boundaries to Hardy space functions on Lipschitz domains. In particular, one can use it to prove Cauchy’s Theorem. See also [6, Lemma 2.7] for the proof.

#### Lemma 2.4

Let $D$ be a bounded Lipschitz domain. Then any $f\in h^1(bD)$ satisfies Cauchy’s Theorem. That is

$$\begin{aligned} \int \limits _{bD} f(\zeta )\, d\zeta = 0\qquad \text {for any}\quad f\in h^1(bD). \end{aligned}$$

Next we state some definitions and results involving Cauchy integrals and the Cauchy transform, which we first define:

#### Definition 2.5

Let $f:bD\rightarrow \mathbb {C}$. The **Cauchy integral** $\textbf{C}_D f$ of *f* is

$$\begin{aligned} \textbf{C}_Df(z):= \frac{1}{2\pi i}\int \limits _{bD}\frac{f(\zeta )}{\zeta - z}\, d\zeta ,\quad z\in D. \end{aligned}$$

Similarly

$$\begin{aligned} \textbf{C}_{\overline{D}^c} f(z):= \frac{1}{2\pi i}\int \limits _{bD}\frac{f(\zeta )}{\zeta - z}\, d\zeta ,\quad z\in \overline{D}^c. \end{aligned}$$

Finally, the **Cauchy transform** $\mathcal C_{D}f$ of *f* is denoted by

$$\begin{aligned} \mathcal C_{D}f(\zeta ):= \dot{(\textbf{C}_{D}f)}(\zeta ),\quad \zeta \in bD. \end{aligned}$$

In both integrals $bD$ is oriented counterclockwise (that is, in the positive direction for $D$).

In this paper we will use the fact that a function *f* in $L^p(bD,\sigma )$ lies in $h^p(bD, \sigma )$ if and only if the Cauchy integral of *f* vanishes on $\overline{D}^c$. This latter fact is well-known for domains with smooth boundaries; here we prove it for Lipschitz domains, see Lemma 2.6 below. We first recall the Plemelj formulas for $f\in L^p(bD,\sigma )$, $1<p< \infty$:

3 $$\begin{aligned} \mathcal C_{D}f(\zeta ) = \frac{1}{2}f (\zeta )+ \frac{1}{2}\mathcal{H}\mathcal{C}_{bD}f(\zeta ), \quad \text{ for } \sigma \text{-a.e. } \zeta \in bD, \end{aligned}$$

and

4 $$\begin{aligned} \lim _{{\mathop { z\in \Gamma (\zeta ,\overline{ D}^c)}\limits ^{z\rightarrow \zeta }}} \textbf{C}_{\overline{D}^c} f(z) = -\frac{1}{2}f(\zeta ) + \frac{1}{2}\mathcal{H}\mathcal{C}_{bD}f(\zeta ) \text{ for } \sigma \text{-a.e. } \zeta \in bD. \end{aligned}$$

Here

$$\begin{aligned} \mathcal{H}\mathcal{C}_{bD} f(\zeta ) := \frac{1}{2\pi i}\,\text {P.V.}\!\!\int \limits _{bD}\frac{f(w)}{w - \zeta }\, dw,\quad \text {for}\ \sigma \text {-a.e.}\ \ \zeta \in bD, \end{aligned}$$

with $bD$ oriented counterclockwise, “P.V.” stands for “principal value,” and $\Gamma (\zeta ,\overline{ D}^c)$ is defined as in Definition 2.2, with *D* in there replaced by $\overline{ D}^c$. Note that a Lipschitz domain $D$ satisfies the exterior cone condition (see [7]) so the limit in (4) is well-defined. A deep result of Coifman, McIntosh, and Meyer [3] states that on bounded Lipschitz domains, $\mathcal{H}\mathcal{C}_{bD}$ is indeed well-defined (i.e. the principal value integral exists $\sigma$-a.e.) and is bounded on $L^p(bD,\sigma )$, $1<p<\infty$. Thus, by the result of [2], the Plemelj formulas (3) and (4) hold (for more on Plemelj formulas, also see [9]).

#### Lemma 2.6

Let $D$ be a bounded simply connected Lipschitz domain and $1<p<\infty$. Assume $f\in L^p(bD,\sigma )$. Then $f\in h^p(bD, \sigma )$ if and only if $\textbf{C}_{\overline{D}^c} f(z) = 0$ for all $z\in \overline{D}^c$.

#### Proof

First assume that $\textbf{C}_{\overline{D}^c} f(z) = 0$ for all $z\in \overline{D}^c$. By Eq. (4), we have

$$0=\lim _{{\mathop { z\in \Gamma (\zeta ,\overline{D}^c)}\limits ^{z\rightarrow \omega }}} C_{\overline{D}^c} f(z) = \frac{1}{2\pi i}\mathrm {P.V.}\int \limits _{bD} \frac{f(\zeta )}{\zeta -\omega }d\zeta -\frac{1}{2}f(\omega ).$$

That is, $\frac{1}{2}f(\omega )= \frac{1}{2\pi i}\mathrm {P.V.}\int \limits _{bD} \frac{f(\zeta )}{\zeta -\omega }$ for $\sigma$-a.e. $\omega \in bD$. Now, using Eq. (3), we have for $\sigma$-a.e. $\omega \in bD$,

$$\begin{aligned} \mathcal C_{D}f(\omega ) = \frac{1}{2\pi i}\mathrm {P.V.}\int \limits _{bD} \frac{f(\zeta )}{\zeta -\omega }d\zeta +\frac{1}{2}f(\omega ) = \frac{1}{2}f(\omega )+\frac{1}{2}f(\omega ) = f(\omega ). \end{aligned}$$

Thus, *f* is in the range of the Cauchy transform. Since the range of the Cauchy transform equals $h^p(bD, \sigma )$ when $D$ is bounded and simply connected and $1<p<\infty$ (see [8]), the backward direction is proven. For the forward direction suppose $f\in h^p(bD, \sigma )$. Then there exists $F\in \mathcal {H}^p(D)$ such that $\dot{F}=f$. Let $z\in \overline{D}^c$ be arbitrary and consider the function $G_z(w):=(w-z)^{-1}$. Then $G_z$ is holomorphic on $D$ and is continuous on $\overline{D}$. Moreover, $\Vert (FG_z)^*\Vert _{L^p(bD,\sigma )}\le \Vert F^*\Vert _{L^p(bD,\sigma )}\Vert G_z^*\Vert _{L^\infty (bD,\sigma )}<\infty$. Thus $FG_z\in \mathcal {H}^p(D)$ and by Cauchy’s Theorem (Lemma 2.4) we have

$$\begin{aligned} 0 = \int \limits _{bD} \dot{(FG_z)}(\zeta ) d\zeta = \textbf{C}_{\overline{D}^c} f(z), \end{aligned}$$

as desired.$$\square $$

## Properties of $\mathcal {H}_{\alpha }^{1,2} (D)$ for Simply Connected $D$

In this section we show that $\mathcal {H}_{\alpha }^{1,2}(D)$ is a reproducing kernel Hilbert space and that it is a subset of the Dirichlet space.

### $\mathcal {H}_{\alpha }^{1, 2}(D)$ is a Reproducing Kernel Hilbert Space

#### Theorem 3.1

Let $D$ be a bounded simply connected Lipschitz domain. Then for any base point $\alpha \in D$:

- $\mathcal {H}_{\alpha }^{1, 2}(D)$ is a Hilbert space with inner product
  $$\displaystyle {\langle F, G\rangle _{\mathcal {H}_{\alpha }^{1,2}(D)}:= \langle \dot{(F')}, \dot{(G')}\rangle _{L^2(bD, \sigma )}. }$$
- For any $z\in D$, the pointwise evaluation: $G\mapsto E_z(G):= G(z)$ is a bounded linear functional on $\mathcal {H}_{\alpha }^{1, 2}(D)$. Hence $\mathcal {H}_{\alpha }^{1, 2}(D)$ is a reproducing kernel Hilbert space (RKHS) with reproducing kernel $K_\alpha ^z(\cdot )=K_\alpha (\cdot ,z)$. Namely, for any $z\in D$, we have that
  $$\dot{(K^z_\alpha )'}(\zeta )\equiv \lim _ {{\mathop {w\in \Gamma (\zeta )}\limits ^{w\rightarrow \zeta }}} (K^z_\alpha )'(w)$$
  exists for almost all $\zeta \in bD$ and for $F\in \mathcal {H}_{\alpha }^{1, 2}(D)$ we have
  5 $$\begin{aligned} F(z)=\int _{bD}\! \dot{({F'})}(\zeta )\, \overline{(\dot{K_\alpha ^z})'(\zeta )}\,d\sigma (\zeta ),\ \ z\in D. \end{aligned}$$
- Let $p\ge 2$ and $g\in \mathfrak {n}^{p}(bD)$. Then for any $\alpha \in D$ the solution of the holomorphic Neumann problem (1) with boundary data *g* has the representation $\displaystyle { G_\alpha (z) = i\int \limits _{\zeta \in bD}\!\!\! g(\zeta )\,\overline{T(\zeta )\,\dot{(K^{z}_\alpha )'}(\zeta )}\, d\sigma (\zeta ),\ \ z\in D. }$

#### Proof

To verify (3.1), note that $\langle \cdot , \cdot \rangle _{\mathcal {H}_{\alpha }^{1,2}(D)}$ is a sesquilinear form and $\langle F, F\rangle _{\mathcal {H}_{\alpha }^{1,2}(D)}=\Vert F\Vert _{\mathcal {H}_{\alpha }^{1,2}(D)}^2$. A straightforward argument (whose details can be found in [6, Lemma 3.4]) shows that for $1\le p\le \infty$ the set $\mathcal H_\alpha ^{1, p}(D)$ is a Banach space with the norm defined as

$$\begin{aligned} \Vert F\Vert _{\mathcal H_\alpha ^{1, p}(D)}= \Vert \dot{(F')}\Vert _{L^p(bD,\sigma )}. \end{aligned}$$

Thus $\mathcal {H}_{\alpha }^{1,2}(D)$ is complete under the norm $\Vert \cdot \Vert _{\mathcal {H}_{\alpha }^{1,2}(D)}$, and so $\mathcal {H}_{\alpha }^{1,2}(D)$ is a Hilbert space.

Next we prove (3.1). Fix $z\in D$ and consider the pointwise evaluation operator $E_z$. For any $\alpha \in D$ and a smooth path $\gamma _\alpha ^z\subset D$ that connects $\alpha$ to *z* we have

$$\begin{aligned} |E_z(G)|=|G(z)| = |G(z)-G(\alpha )|=\left| \,\int \limits _{\gamma _\alpha ^z} G'(w)dw\right| \le |\gamma _\alpha ^z| \sup _{w\in \gamma _\alpha ^z} |G'(w)| \, . \end{aligned}$$

Furthermore, for any $w\in \gamma _\alpha ^z$, Cauchy formula and Hölder inequality give

$$\begin{aligned} |G'(w)| = \frac{1}{2\pi } \left| \,\int \limits _{bD} \frac{\dot{(G')}(\zeta )}{w-\zeta }d\zeta \right| \le \frac{ |bD|^{\frac{1}{2}}}{2\pi k_z}\Vert \dot{(G')}\Vert _{L^2(bD,\sigma )}=\frac{ |bD|^{\frac{1}{2}}}{2\pi k_z}\Vert G\Vert _{\mathcal {H}_{\alpha }^{1,2}(D)}, \end{aligned}$$

where $k_z:= {{\,\textrm{dist}\,}}(\gamma _\alpha ^z,bD) >0$. Combining all of the above we see that for any $z\in D$, $E_z$ is a bounded linear functional on $\mathcal {H}_{\alpha }^{1,2}(D)$; Hilbert space theory now grants the existence of the reproducing kernel function

$$K^z_\alpha \in \mathcal {H}_{\alpha }^{1,2}(D)\quad \text {with}\quad G(z)=\langle G, K^z_\alpha \rangle _{\mathcal {H}_{\alpha }^{1,2}(D).}$$

Finally we verify (3.1). Let $p\ge 2$ and $g\in \mathfrak {n}^{p}(bD)$. Suppose $G_\alpha \in \mathcal {H}_{\alpha }^{1,p}(D)$ is the solution to the Neumann problem (1) with datum *g*. Thus $\dot{(G_\alpha ')} =i\overline{T}g$ and $G_\alpha \in \mathcal {H}_{\alpha }^{1,2}(D)$. Hence for any $z\in D$ we have

$$\begin{aligned} G_\alpha (z) = \langle G_\alpha , K^z_\alpha \rangle _{\mathcal {H}_{\alpha }^{1,2}(D)} = \int \limits _{bD}\dot{(G'_\alpha )}(\zeta )\overline{(\dot{K^z_\alpha })'(\zeta )}d\sigma (\zeta ) = i\int \limits _{bD}g(\zeta )\overline{T(\zeta )(\dot{K^z_\alpha })'(\zeta )}d\sigma (\zeta ), \end{aligned}$$

as desired.$$\square $$

In the case of the unit disc $\mathbb {D}$ we obtain explicit formulas and recover the full range of $1\le p\le \infty$:

#### Theorem 3.2

1. The reproducing kernel associated to $\mathcal {H}_{\alpha }^{1,2}(\mathbb D)$ is given by
  6 $$\begin{aligned} K^z_\alpha (w)=\sum _{k=1}^{\infty }\frac{(w^k-\alpha ^k)\overline{(z^k-\alpha ^k)}}{2\pi k^2},\qquad z,w\in \mathbb {D}. \end{aligned}$$
2. Given $g\in \mathfrak {n}^{p}(b\mathbb D)$, $1< p < \infty$ and $\alpha :=0$, the unique solution $G\in \mathcal {H}_0^{1,p}(\mathbb D)$ to the holomorphic Neumann problem (1) admits the following representation
  7 $$\begin{aligned} G(z)=\frac{1}{2\pi }\int \limits _{b\mathbb D}g(\zeta )\textrm{Log}\frac{1}{1-z\overline{\zeta }}\,d\sigma (\zeta ), \end{aligned}$$
  where $\textrm{Log}$ denotes the principal branch of the complex logarithm.

#### Proof

To prove part *1.*, note that since $\mathbb {D}$ is simply connected every holomorphic function on $\mathbb {D}$ has an antiderivative. Thus the mapping $G\mapsto G'$ is an isometric isomorphism from $\mathcal {H}_{\alpha }^{1,2}(\mathbb {D})$ onto $\mathcal {H}^{2}(\mathbb {D})$. Since $\{{\frac{1}{ \sqrt{2\pi }}} z^{k-1}\}_{k\in \mathbb {N}}$ is an orthonormal basis of $\mathcal {H}^{2}(\mathbb {D})$, the set of antiderivatives $\{\frac{z^k-\alpha ^k}{\sqrt{2 \pi } k}\}_{k\in \mathbb {N}}$ is an orthonormal basis of $\mathcal {H}_{\alpha }^{1,2}(\mathbb {D})$. Thus, by the theory of reproducing kernel Hilbert spaces, $K_\alpha$ as given in Eq. (6) is the reproducing kernel for $\mathcal {H}_{\alpha }^{1,2}(\mathbb {D})$.

For the proof of part *2.*, note that the reproducing kernel for $\mathbb {D}$ satisfies

$$\begin{aligned} \overline{(K_0^z)'(w)} =\frac{\partial }{\partial \overline{w}}\sum _{k=1}^{\infty }\frac{z^k{\overline{w}}^k}{2\pi k^2} =\sum _{k=1}^{\infty }\frac{z^k{\overline{w}}^{k-1}}{2\pi k}=\frac{1}{2\pi \overline{w}}\textrm{Log}\frac{1}{1-z\overline{w}}, \quad {w\in \mathbb {D}}. \end{aligned}$$

Hence for every $\zeta \in b\mathbb {D}$, and since $T(\zeta ) = i\zeta$, we have

$$\begin{aligned} \overline{T(\zeta )\dot{(K_0^z)'}(\zeta )} = \frac{1}{2\pi i}\textrm{Log}\frac{1}{1-z\overline{\zeta }}, \quad {\zeta \in b\mathbb {D}}. \end{aligned}$$

So for $g\in \mathfrak {n}^2(b\mathbb {D})$ we have that Eq. (7) follows from the above and Theorem 3.1 part (3.1).

For $g\in \mathfrak {n}^{p}(b\mathbb D)$, $1< p< \infty$, define *G* as in (7). Then $G\in \vartheta (\mathbb D)$ and

$$G'(z) = \frac{1}{2\pi }\int \limits _{b\mathbb D}\frac{g(\zeta ) \bar{\zeta }}{1-z\overline{\zeta }}\,d\sigma (\zeta )$$

$$ = \frac{1}{2\pi }\int \limits _{b\mathbb D}\frac{g(\zeta ) }{\zeta -z }\,d\sigma (\zeta )= \frac{1}{2\pi i} \int \limits _{b\mathbb D}\frac{i\overline{ T(\zeta )} g(\zeta ) }{\zeta -z }\,d \zeta = \textbf{C}_{\mathbb D} (i\overline{T}g)(z),\ \ z\in \mathbb D. $$

Here we used the facts that $\zeta \bar{\zeta }=1$ and $d\sigma (\zeta ) = \overline{ T(\zeta ) } d\zeta$ on $\mathbb D$. Consequently, $(G')^*\in L^p(b\mathbb D, \sigma )$ by a mapping property of the Cauchy integral $\textbf{C}_{\mathbb D}$ and Cauchy transform $\mathcal C_{\mathbb D}$ (see [8, Lemma 2.12], noting that the assumption that $p\ne 1, \infty$ is needed here). Moreover, from the above we also have that

$$\dot{(G')}(\zeta )= \mathcal C_{\mathbb D} (i\overline{T}g)(\zeta ), \quad \text{ a.e. } \zeta \in b\mathbb {D}.$$

But $\overline{T}g\in h^p(b\mathbb D)$ because $g\in \mathfrak {n}^p(b\mathbb {D})$, and $\mathcal C_{\mathbb D}$ is the identity on $h^p(b\mathbb D)$, thus

$$\frac{\partial G}{\partial n}(\zeta ) =-iT(\zeta ) \dot{(G')}(\zeta ) = -iT(\zeta )\mathcal C_{\mathbb D}(i\overline{T}g )(\zeta ) = g(\zeta ), \ \ \zeta \in b\mathbb D\ \ \sigma -a.e.$$

That is, *G* solves (1) for $1< p < \infty$. (Uniqueness was proved in [6].)

### $\mathcal {H}_{\alpha }^{1,p}(D)$ is Embedded in the Dirichlet Space

In [1], Axler and Shields introduced the **Dirichlet space** $\mathcal {D}^2_{\alpha }(D)$ for a general domain $D$, namely

$$\begin{aligned} \mathcal {D}^2_{\alpha }(D):=\left\{ F\in \vartheta (D):F(\alpha )=0,\, \int \limits _{D}\left| F'(z)\right| ^2\,dV(z)<\infty \right\} ,\quad \alpha \in D, \end{aligned}$$

which is a Hilbert space with inner product

$$\langle F,G\rangle _{\mathcal {D}^2_{\alpha }(D)}:= \int \limits _{D} F'(z)\,\overline{G'(z)}\, dV(z).$$

(Here *dV* is the Lebesgue measure for $\mathbb C$.) The analogous definition of $\mathcal {D}^p_{\alpha }(D)$ with $1\le p\le \infty$ yields a Banach space with norm

$$ \Vert F\Vert _{\mathcal {D}^p_{\alpha }(D)}:= \int \limits _D|F'(z)|^p\, dV (z). $$

#### Theorem 3.3

Let $D$ be a bounded simply connected Lipschitz domain and $1<p<\infty$. Suppose that $F\in \mathcal {H}_{\alpha }^{1,p}(D)$. Then $F\in \mathcal {D}^p_{\alpha }(D)$ and

$$ \Vert F\Vert _{\mathcal {D}^p_{\alpha }(D)}\lesssim \Vert F\Vert _{\mathcal {H}_{\alpha }^{1,p}(D)}.$$

That is, the holomorphic Sobolev–Hardy space is embedded in the Dirichlet space.

To prove Theorem 3.3 we need the following result:

#### Lemma 3.4

Let $D$ be a bounded simply connected Lipschitz domain and $1< p<\infty$. Suppose that $F\in \mathcal {H}^p(D)$. Then $F\in \vartheta (D)\cap L^p(D)$ and

$$ \Vert F\Vert _{L^p(D)}\lesssim \Vert \dot{F}\Vert _{L^p(bD, \sigma )}. $$

That is, the holomorphic Hardy space is embedded in the Bergman space.

#### Proof

In [6, Lemma 2.8] it is shown that if $1<p<\infty$ and $D$ is a simply connected and bounded Lipschitz domain, then for $F\in \mathcal {H}^p(D)$ quantities $\Vert F^*\Vert _{L^p(bD, \sigma )}$ and $\Vert \dot{F}\Vert _{L^p(bD, \sigma )}$ are comparable. Thus it suffices to show that $\Vert F\Vert _{L^p(D)}\lesssim \Vert F^*\Vert _{L^p(bD, \sigma )}$.

Consider a Nečas exhaustion $\{D_k\}$ of $D$. Then there are finitely many coordinate rectangles $R_j:=[a_j,b_j]\times (c_j,d_j)$ with Lipschitz functions $\phi ^j_k$ and $\phi ^j$ whose graphs determine $D_k$ and $D$, respectively, on $R_j$ and $\phi ^j_k$ converges uniformly to $\phi ^j$. For any $k\in \mathbb {N}$, $x\in [a_j,b_j]$ and $y\in \left( \phi ^j(x),\phi ^j_k(x)\right]$, the point $x+iy$ lies directly above $x+i\phi ^j(x)$ and thus $x+iy\in \Delta _1 + (x+i\phi ^j(x))$, where $\Delta _1$ is the cone in Definition 2.2. And so $e^{-i\theta _j}(x+iy)$ lies in $e^{-i\theta _j}(\Delta _1+ (x+i\phi ^j(x)))\subseteq \Gamma (e^{-i\theta _j}(x+i\phi ^j(x)))$. Fix $k\in \mathbb {N}$ so that for each *j* we have $\Vert \phi ^j_k-\phi ^j\Vert _{\infty }<1$. Then we have for $F\in \mathcal {H}^p(D)$

$$\begin{aligned} \iint _{D-D_k} |F(z)|^p dA(z)\le &   \sum _{j} \iint _{e^{-i\theta _j}R_j\cap (D-D_k)} |F(z)|^p dA(z) \\= &   \sum _{j} \int \limits _{a_j}^{b_j} \int \limits _{\phi ^j(x)}^{\phi _k^j(x)} |F(e^{-i\theta _j}(x+iy))|^p dy dx\\\le &   \sum _{j} \int \limits _{a_j}^{b_j} \int \limits _{\phi ^j(x)}^{\phi _k^j(x)} F^*(e^{-i\theta _j}(x+i\phi _{j}(x)))^p dy dx\\\le &   \sum _{j} \int \limits _{a_j}^{b_j} F^*(e^{-i\theta _j}(x+i\phi _{j}(x)))^p dx\\\le &   \sum _{j} \int \limits _{a_j}^{b_j} F^*(e^{-i\theta _j}(x+i\phi _{j}(x)))^p \left| e^{i\theta _j}(1+i\phi _j'(x))\right| dx \\= &   \sum _{j} \int \limits _{bD\cap e^{-i\theta _j}R_j} F^*(\zeta )^p d\sigma (\zeta ) \lesssim \Vert F^*\Vert _{L^p(bD,\sigma )}^p. \end{aligned}$$

Since $D_k$ is compactly contained in $D$ and *k* is fixed, ${{\,\textrm{dist}\,}}(D_k, bD)>d$ for some constant *d* depending on $D$. So, similar to the argument of the proof of part (3.1) of Theorem 3.1, by the Cauchy integral formula we have

$$\iint _{D_k} |F(z)|^p dA(z) \lesssim \Vert F^*\Vert _{L^p(bD,\sigma )}^p,$$

completing the proof to $\Vert F\Vert _{L^p(D)}\lesssim \Vert F^*\Vert _{L^p(bD, \sigma )}$. $\square$

#### Proof of Theorem 3.3

Let $F\in \mathcal {H}_{\alpha }^{1,p}(D)$. Then $F(\alpha )=0$ and $F'\in \mathcal {H}^p(D)$. By Lemma 3.4, we also have $F'\in \vartheta (D)\cap L^p(D)$ giving that $F\in \mathcal {D}^p_\alpha (D)$, as desired.$\square$

## Characterizations of $\mathfrak {n}^{p}(bD)$ for Simply Connected $D$

### Theorem 4.1

Let $D$ be a bounded simply connected Lipschitz domain and $1\le p\le \infty$. Then $\mathfrak {n}^{p}(bD)$ defined as in (2) is closed in the $L^p(bD, \sigma )$-norm. Moreover, for

$$\begin{aligned} \mathfrak {n}_1:= &   \{Tg:g\in h^p(bD)\},\\ \mathfrak {n}_2:= &   \left\{ f\in L^p(bD, \sigma ): \int \limits _{bD} \zeta ^k f(\zeta ) d\sigma (\zeta ) = 0 \text{ for } \text{ all } k=0,1,2,\ldots \right\} ,\\ \mathfrak {n}_3:= &   \left\{ f\in L^p(bD,\sigma ): \textbf{C}_{\overline{D}^c} (\overline{T}f)= 0\right\} \end{aligned}$$

we have that $\mathfrak {n}^{p}(bD)=\mathfrak {n}_1=\mathfrak {n}_2$. If $1<p<\infty$, then we also have $\mathfrak {n}^{p}(bD)=\mathfrak {n}_3$.

### Proof

The inclusion $$\mathfrak {n}^{p}(bD)\subseteq \mathfrak {n}_1$$ is immediate from (2). The reverse inclusion holds because $$D$$ is simply connected and thus all holomorphic functions on $$D$$ have antiderivatives. As $$h^p(bD)$$ is closed in the $$L^p(bD, \sigma )$$-norm, we see that $$\mathfrak {n}_1$$, and thus $$\mathfrak {n}^{p}(bD)$$ is also closed. Next, the identity $$\mathfrak {n}^{p}(bD)=\mathfrak {n}_2$$ follows from the fact that $$T(\zeta )d\sigma (\zeta )=d\zeta $$ and the well-known result of Smirnov that $$g\in L^p(bD,\sigma )$$ lies in $$h^p(bD) $$ if and only if

$$\int \limits _{bD} \zeta ^k g(\zeta ) d\zeta = 0 \ \ \text { for}\ \ k=0,1,2,\ldots $$

See, for example, [5, Theorem 10.4]. Finally, the identity $\mathfrak {n}^{p}(bD)=\mathfrak {n}_3$ for $1<p<\infty$ follows from Lemma 2.6.$\square$

We may also characterize the elements of $\mathfrak {n}^p(bD)$ for a bounded simply connected Lipschitz domain $D$ via its Riemann maps. We shall need the following description of the tangent vector.

### Lemma 4.2

Let $D$ be a bounded simply connected Lipschitz domain and $\psi :D\rightarrow \mathbb {D}$ be a conformal map. Then the tangent vector *T* of $bD$ (which is defined a.e.) can be written as

$$\begin{aligned} T = i\frac{|\dot{(\psi ')}|}{\dot{(\psi ')}}\dot{\psi }, \ \ \ \ \sigma \text {-a.e. on}\ \ bD. \end{aligned}$$

### Proof

Let $\phi :\mathbb {D}\rightarrow D$ be defined as $\phi =\psi ^{-1}$. Since $bD$ is Lipschitz, it is a Jordan curve so by Carathéodory’s theorem $\phi$ extends to a homeomorphism of $\overline{\mathbb {D}}$ onto $\overline{D}$. Thus $\psi$ extends to a homeomorphism of $\overline{D}$ onto $\overline{\mathbb {D}}$ and that extension restricted to $bD$ must equal $\dot{\psi }$. By [5, Theorem 3.13], we have that $\phi '\in \mathcal {H}^1(\mathbb {D})$ so that $\dot{(\phi ')}$ exists $\sigma$-a.e., $\phi$ is absolutely continuous on $b\mathbb {D}$, and

8 $$\begin{aligned} \frac{d}{dt}\phi (e^{it})=ie^{it}\dot{(\phi ')}(e^{it}). \end{aligned}$$

Thus we can write the unit tangent vector *T* via $\dot{(\phi ')}$ for almost all $\zeta \in \partial D$. To do so, first note that for $r<1$

$$\begin{aligned} \psi '(\phi (re^{it}))=\frac{1}{\phi '(re^{it})}. \end{aligned}$$

Since $\phi$ is conformal and $\dot{(\phi ')}$ exists and is nonzero a.e., we see that the nontangential limit $\dot{(\psi ')}$ exists a.e. and satisfies

9 $$\begin{aligned} \dot{(\psi ')}(\zeta )=\frac{1}{\dot{(\phi ')}(\dot{\psi }(\zeta ))}. \end{aligned}$$

Choose $t_0$ so that $\zeta =\phi (e^{it_0})$. Then by Eqs. (8) and (9) we have

$$\begin{aligned} T(\zeta )= &   \left. \frac{\frac{d}{dt}\phi (e^{it})}{|\frac{d}{dt}\phi (e^{it})|}\right| _{t=t_0}\\= &   \frac{\dot{(\phi ')}(e^{it_0})ie^{it_0}}{|\dot{(\phi ')}(e^{it_0})|}=i\frac{\dot{(\phi ')}(\psi (\zeta ))\dot{\psi }(\zeta )}{|\dot{(\phi ')}(\psi (\zeta ))|}=i\frac{|\dot{(\psi ')}(\zeta )|\dot{\psi }(\zeta )}{\dot{(\psi ')}(\zeta )}\sigma \text{-a.e. }, \end{aligned}$$

as desired.$$\square $$

### Theorem 4.3

Let $D$ is a bounded simply connected Lipschitz domain, and $1\le p\le \infty$. Let $\psi :D\rightarrow \mathbb {D}$ be a conformal map with $\alpha : =\psi ^{-1}(0)\in D$. Then

$$\mathfrak {n}^p(bD) = \left\{ \frac{\dot{(\psi ')}}{|\dot{(\psi ')}|} \dot{F}: F\in \mathcal H^p(D), F(\alpha )=0\right\} . $$

### Proof

First by Proposition , one has

$$\mathfrak {n}^p(bD) = \left\{ T\dot{G}: G\in \mathcal H^p(D) \right\} . $$

Making use of Lemma 4.2, we further obtain

$$\mathfrak {n}^p(bD) = \left\{ \frac{\dot{(\psi ')}}{|\dot{(\psi ')}|} \dot{\psi }\dot{G}: G\in \mathcal H^p(D) \right\} . $$

Note that $\psi$ is conformal on $D$ and continuous on $\overline{D}$. In particular, $\psi$ has only one zero at $\alpha$ and that zero is simple. Letting $F: = \psi G$, then

$$ G \in \mathcal H^p(D) \ \ \text { if and only if }\ \ F \in \mathcal H^p(D), F(\alpha )=0. $$

The proof is complete.$\square$

Note that for $D=\mathbb {D}$ we can choose $\psi (z)=z$, in which case Theorem 4.3 takes an especially simple form, namely

$$ \mathfrak {n}^p(b\mathbb {D}) = \{\dot{F}: F\in \mathcal H^p(\mathbb {D}), F(0)=0\}. $$

## A Characterization of $\mathfrak {n}^p(bD)$ for Multiply Connected $D$

Let $D$ be a bounded Lipschitz domain. Then there exists $N\ge 1$, such that the boundary $bD$ consists of *N* closed rectifiable curves. Here and throughout we denote by $\gamma _1, \gamma _2,\ldots ,\gamma _N$ those closed curves of $bD$ endowed with the positive orientation, with $\gamma _N$ denoting the outer curve of $bD$ (that is, $D$ lies in the set of points inside of $\gamma _N$).

In order to characterize $\mathfrak {n}^p(bD)$ we need to understand which elements of $\mathcal H^p(D)$ admit holomorphic antiderivatives. According to classical complex analysis theory, a continuous complex-valued function has an antiderivative in a domain $D$ (which may be simply or multiply-connected) if and only if the line integral of the function along every closed contour (i.e. piecewise $C^1$ path) in $D$ is zero. See, for instance, [11, Thereom 6.44]. This leads us to the following:

### Proposition 5.1

Let $D$ be a bounded Lipschitz domain and let the boundary of $D$ be denoted as above. For $1\le p\le \infty$ and $F\in \mathcal {H}^p(D)$ we have that *F* is the complex derivative of a holomorphic function on $D$ if and only if

10 $$\begin{aligned} \int \limits _{\gamma _j} \dot{F}(\zeta ) d\zeta =0\quad \text{ for } \text{ all } \quad j = 1, \ldots , N\text{. } \end{aligned}$$

### Proof

Let $\{D_k\}$ be Nečas exhaustion of $D$ as defined in Lemma 2.3. We will use the notation of Lemma 2.3 throughout this proof. For each *k* and $1\le j\le N$, let $\gamma ^k_j$ denote portion of $bD_k$ such that $\Lambda _k(\gamma ^k_j)=\gamma _j$.

First, assume *F* is a derivative of a holomorphic function on $D$. For each *k* the curve $\gamma ^k_j$ is a closed contour in $D$. Thus, by the Fundamental Theorem of Calculus, we have

$$ \int \limits _{\gamma ^k_j} F(\zeta ) d\zeta =0,\quad j=1, \ldots , N. $$

Thus

11 $$\begin{aligned} 0= &   \lim _{k\rightarrow \infty }\int \limits _{\gamma ^k_j} F(\zeta ) d\zeta = \lim _{k\rightarrow \infty }\int \limits _{\gamma ^k_j} F(\eta _k)T_k(\eta _k)d\sigma _k(\eta _k)\nonumber \\= &   \lim _{k\rightarrow \infty }\int \limits _{\gamma _j} F(\Lambda _k(\eta ))T_k(\Lambda _k(\eta ))w_k(\eta )d\sigma (\eta ) = \int \limits _{\gamma _j} \dot{F}(\eta )T(\eta )d\sigma (\eta ) = \int \limits _{\gamma _j} \dot{F}(\zeta )d\zeta ,\nonumber \\ \end{aligned}$$

where we used the Dominated Convergence Theorem with the dominating function $M|F^*|$ (here we are using the fact that $F\in \mathcal {H}^p(D)$ so that $F^*\in L^1(bD,\sigma )$), obtaining (10).

Conversely, assume (10) holds. Fixing a point $a\in D$, we shall show that for any $z\in D$, and any contour $\eta$ in $D$ connecting *a* and *z*, the following line integral

$$ \int \limits _{\eta } F(\zeta ) d\zeta $$

is independent of the choice of the path.

Indeed, let $\eta _1$ and $\eta _2$ be two contours joining *a* and *z* and let $\beta =\eta _1\cup (-\eta _2)$ be the closed contour starting and ending at *a* (here $-\eta _2$ is $\eta _2$ oriented in the opposite direction). Without loss of generality, suppose $\beta$ is oriented counterclockwise and has no self-intersections. If the domain bounded by $\beta$ is a subset of $D$, then $\int _\beta F(\zeta )d\zeta =0$ by Cauchy’s theorem. Else, for some *m* between 1 and *N* there are *m* components of $bD$, say, $\gamma _{1},\ldots ,\gamma _{m}$, that lie inside the domain bounded by $\beta$, while the remaining components $\gamma _{m+1},\ldots ,\gamma _{N}$ lie outside of such domain. With same notation as before, for a Nečas exhaustion $\{D_k\}$, we choose *k* large enough so that $D_k$ contains $\beta$, $\gamma ^k_{1},\ldots ,\gamma ^k_{m}$ lie inside of $\beta$, and $\gamma ^k_{m+1},\ldots ,\gamma ^k_{N}$ lie outside of $\beta$. By a generalized version of Cauchy’s theorem (see, for example, [11, Thereom 8.9]),

$$ \int _\beta F(\zeta )d\zeta = \sum _{\ell =1}^m\int _{\gamma ^k_{\ell }} F(\zeta )d\zeta \quad \text{ for } \text{ any } \text{ large } \text{ k. }$$

By an argument similar to the proof of Eq. (11) we have

$$ \int _\beta F(\zeta )d\zeta = \lim _{k\rightarrow \infty } \sum _{\ell =1}^m\int _{\gamma ^k_{\ell }} F(\zeta )d\zeta = \sum _{\ell =1}^m\int _{\gamma _{\ell }} \dot{F}(\zeta )d\zeta =0,$$

where we used (10) in the last equality. Equivalently,

$$ \int \limits _{\eta _1} F(\zeta ) d\zeta = \int \limits _{\eta _2} F(\zeta ) d\zeta ,$$

thus

$$H(z): = \int \limits _{\eta } F(\zeta ) d\zeta $$

is well defined and is a holomorphic antiderivative of *F* on $D$.$\square$

### Remark 5.2

By Cauchy’s theorem (in Lemma 2.4), we have

$$\begin{aligned} \sum _{j=1}^N \int \limits _{\gamma _j} \dot{F}(\zeta ) d\zeta = \int \limits _{bD} \dot{F}(\zeta ) d\zeta =0, \end{aligned}$$

for any $F\in \mathcal {H}^p(D)$. Then we can refine the statement of Proposition 5.1 by requiring that only $(N-1)$-many terms in Eq. (10) vanish. Without loss of generality, we choose the first $(N-1)$ terms. Hence, Eq. (10) is equivalent to

12 $$\begin{aligned} \int \limits _{\gamma _j} \dot{F}(\zeta ) d\zeta =0\quad \text{ for } \text{ all } \quad j = 1, \ldots , N-1. \end{aligned}$$

### Theorem 5.3

Let $D$ be a bounded Lipschitz domain and $1\le p\le \infty$. Then with $\mathfrak {n}^{p}(bD)$ as in (2) we have

13 $$\begin{aligned} \mathfrak {n}^{p}(bD)= \left\{ Tf:\ f\in h^p(bD),\quad \int \limits _{\gamma _j}\!\! f(\zeta )\,d\zeta = 0, \quad 1\le j\le N-1\right\} . \end{aligned}$$

If $D$ is simply connected then the above identity reads $\mathfrak {n}^{p}(bD)= \mathfrak {n}_1$, see Theorem (we should perhaps point out that the congruence of $\mathfrak {n}^p(bD)$ with the two spaces $\mathfrak {n}_2$ and $\mathfrak {n}_3$ proved therein relies upon results that are classically stated for simply connected $D$).

### Proof

Let

$$ L_0^p(bD, \sigma ): =\left\{ g \in L^p(bD, \sigma ):\int \limits _{bD} \!\! g(\zeta ) d\sigma (\zeta ) =0\right\} $$

and

$$L_{00}^p(bD, \sigma ): =\left\{ g\in L^p(bD,\sigma ): \ \int \limits _{\gamma _j} \!\! g(\zeta ) d\sigma (\zeta ) =0, \quad 1\le j\le N\right\} .$$

Obviously $L_{00}^p(bD, \sigma )\subset L_{0}^p(bD, \sigma )$. We claim that

14 $$\begin{aligned} \mathfrak {n}^{p}(bD)= \{ g\in L_{00}^p(bD, \sigma ): \overline{T} g\in h^p(bD) \}. \end{aligned}$$

Indeed, if $g\in \mathfrak {n}^{p}(bD)$ there exists a $G\in \vartheta (D)$ with $G'\in \mathcal {H}^p(D)$ such that $g= -iT\dot{( G')}$, see (2); hence $\overline{T} g = -i\dot{( G')}\in h^p(bD)$. Moreover Proposition 5.1 gives that

$$\begin{aligned} \int \limits _{\gamma _j} g(\zeta ) d\sigma (\zeta )= -i\int \limits _{\gamma _j} \dot{(G')}(\zeta )d\zeta =0,\quad j=1,\ldots , N-1 \end{aligned}$$

proving that $g\in {L}_{00}^p(bD,\sigma )$ and concluding the proof of the forward inclusion. For the reverse inclusion, suppose $g\in L_{00}^p(bD, \sigma )$ and $g=T\dot{F}$ for some $F\in \mathcal {H}^p(D)$. Then

$$\begin{aligned} \int \limits _{\gamma _j} \dot{F}(\zeta )d\zeta = \int \limits _{\gamma _j} g(\zeta )d\sigma (\zeta )=0,\quad j=1,\ldots , N-1 \end{aligned}$$

and it follows from Proposition 5.1 and Eq. (12) that *F* has an antiderivative $G\in \vartheta (D)$. Note that $iG\in \mathcal {H}^{1, p}(D)$ by definition. Thus, $g=T\dot{F}=-iT(\dot{iG'})\in \mathfrak {n}^{p}(bD)$. The proof of (14) is concluded. Equation (13) now follows since for *g* as above we have $g=Tf$ with $f:=\overline{T} g$.$\square$

## Data Availability

No datasets were generated or analysed during the current study.
